# Peroxisome Proliferator-Activated Receptors (PPARs) in Psoriasis: Metabolic Intersections, Molecular Mechanisms, and Potential Treatments

**DOI:** 10.7759/cureus.104583

**Published:** 2026-03-02

**Authors:** Venkata Bharat Kumar Pinnelli, Surendra Babu T, Jayashankar CA, Prakash Bhanu, Aga Ammar Murthuza, Mansi Thipani Madhu, Srilekha N, Koshy T Sam, Mir Hyder Hussain, Akshay AS, Venkataramana Kandi

**Affiliations:** 1 Biochemistry, Vydehi Institute of Medical Sciences and Research Centre, Bengaluru, IND; 2 Anatomy, Vydehi Institute of Medical Sciences and Research Centre, Bengaluru, IND; 3 Internal Medicine, Vydehi Institute of Medical Sciences and Research Centre, Bengaluru, IND; 4 Dermatology, Vydehi Institute of Medical Sciences and Research Centre, Bengaluru, IND; 5 Medicine, Vydehi Institute of Medical Sciences and Research Centre, Bengaluru, IND; 6 General Medicine, Vydehi Institute of Medical Sciences and Research Centre, Bengaluru, IND; 7 Clinical Microbiology, Prathima Institute of Medical Sciences, Karimnagar, IND

**Keywords:** anti-psoriatic efficacy, metabolic homeostasis, metabolic syndrome, peroxisome proliferator-activated receptors (ppars), ppar modulation, psoriasis, treatment approach

## Abstract

Psoriasis is a chronic, systemic inflammatory disease characterized by combined epidermal and immunological pathology. It is becoming recognized as a metabolic inflammatory disorder caused by interleukin (IL)-23/Th17 axis imbalance and lipid metabolism dysfunction. Peroxisome proliferator-activated receptors (PPARs), including peroxisome proliferator-activated receptor alpha (PPARα), peroxisome proliferator-activated receptor beta/delta (PPARβ/δ), and peroxisome proliferator-activated receptor gamma (PPARγ), regulate metabolic homeostasis and immune tolerance, making them potential targets for treating the *psoriatic march* of comorbidities such as obesity, insulin resistance, psoriatic arthritis, non-alcoholic fatty liver disease, and accelerated atherosclerosis. This review aims to summarize current evidence on PPAR isoform-specific roles in psoriatic pathogenesis, explore the molecular mechanisms that link PPARs to cutaneous and systemic inflammation, and assess the therapeutic potential and translational challenges of PPAR-directed pharmacotherapy. Peer-reviewed research on PPAR biology in psoriasis, metabolic comorbidities, and the clinical effectiveness of PPAR ligands was identified by a comprehensive literature search of PubMed/MEDLINE, Scopus, and Web of Science (2014-2024, with seminal papers from 2000+). Real-world evidence, randomized controlled trials, and mechanistic studies were given priority in the analysis. In psoriatic lesions, PPARγ is downregulated, leading to nuclear factor-κB and signal transducer and activator of transcription 3 deregulation. This promotes keratinocyte hyperproliferation and Th17 cell differentiation. PPARβ/δ is overexpressed, leading to anaerobic glycolysis and peroxisomal fatty acid β-oxidation. This depletes structural barrier lipids and maintains hyperplasia. Reduced PPARα inhibits lipogenesis at the epidermal barrier. Thiazolidinedione agonists (pioglitazone, rosiglitazone) have modest but clinically significant anti-psoriatic efficacy when combined with traditional systemic medicines, resulting in cardiometabolic benefits. Preclinical studies suggest that PPAR-selective antagonists, such as 4-chloro-N-(2-{[5-trifluoromethyl)-2-pyridyl] sulfonyl} ethyl)benzamide 3 (GSK3787) for PPARδ, outperform broad agonism. Topical PPAR ligand bioavailability remains inadequate, necessitating innovative delivery strategies. Although PPAR modulation offers a dual-benefit treatment approach that simultaneously suppresses metabolic dysregulation and cutaneous inflammation, there are still significant gaps between genetic potential and clinical reality. Future directions include patient biomarker stratification, dual/selective agonists (glitazars), and sensible combination with biologics that target tumor necrosis factor-alpha/IL-17/IL-23. This review reframes PPARs as key players in the relationship between psoriasis and metabolic syndrome by synthesizing molecular understanding and clinical data.

## Introduction and background

Psoriasis affects 2-3% of the global population and is much more than just a skin condition [1,2]. The disease is characterized by chronic, thymus (T)-derived, lymphocyte cell-driven inflammation that causes abnormal keratinocyte differentiation and hyperproliferation, but its systemic manifestations, such as psoriatic arthritis (which affects 30% of psoriasis patients), metabolic syndrome (MeS), obesity, insulin resistance (IR), non-alcoholic fatty liver disease (NAFLD), and accelerated atherosclerosis (ATS), highlight its nature as a systemic immune-mediated condition [3-5]. Patients with psoriasis face up to a 50% higher risk of cardiovascular disease, with severe cases tripling the odds of heart attack and raising the risks of stroke and cardiovascular death by 60% and 40%, respectively. Multiple studies have shown that psoriasis increases all-cause mortality by 21%, with notable rises in deaths from cardiovascular (40%), infectious (50%), and renal diseases (70%). Each decade with psoriasis boosts vascular inflammation by 41%, resulting in a 58% higher rate of major cardiovascular events such as heart attacks and strokes. These findings highlight the importance of comprehensive management beyond dermatology [6-8]. This increased cardiovascular risk is proportional to the severity of the skin condition, and treating cutaneous inflammation has been linked to reduced vascular inflammation, indicating similar inflammatory pathways [9,10].

The current illness model prioritizes the interleukin (IL)-23/T-helper (Th) 17 axis as the primary immunopathogenic driver, transcending previous Th1-centric paradigms [2,11]. IL-23, primarily produced by dendritic cells and keratinocytes, promotes Th17 cell differentiation and expansion, which, in turn, secrete IL-17A, IL-17F, and IL-22, collectively driving keratinocyte activation, antimicrobial peptide production, and further amplification of innate and adaptive inflammatory circuits [12,13]. Biological therapy targeting IL-23p19, IL-17A, and tumor necrosis factor-alpha (TNF-α) confirms the concept. However, the variety of biologic responses and the formation of secondary resistance imply that pathway targeting alone is insufficient [14,15].

Psoriasis is becoming recognized as a metabolic-inflammatory nexus, rather than only an inflammatory immunological disease. Psoriasis patients have systemic metabolic derangements, including increased adiposity, adipokine dysregulation (elevated TNF-α, IL-6, leptin; reduced adiponectin), IR, dyslipidemia, and poor lipid barrier homeostasis in the skin [16-19]. Psoriasis, affecting 2-3% of the global population, is increasingly linked to systemic metabolic dysfunction. Up to 40% of patients develop MeS, with elevated risks of obesity, diabetes, and cardiovascular disease [20]. Obesity amplifies psoriasis severity, with 25% of patients obese, and each unit increase in relative fat mass raises psoriasis odds by ≈7%. Visceral fat (waist‑to‑hip ratio OR ≈ 1.26) strongly correlates with disease risk, driving metabolic inflammation, biologic resistance, and heightened cardiovascular comorbidity [21]. Transcriptome analyses reveal keratinocytes as active contributors to psoriasis pathogenesis. Differential expression identified over 1,000 genes, with ≈300 upregulated inflammatory mediators and ≈200 downregulated regulatory pathways. These dynamic changes highlight the role of keratinocytes in amplifying immune responses, metabolic dysfunction, and chronic inflammation, reinforcing psoriasis as a systemic disease [22]. This new perspective shows that treatment strategies that address both inflammation and metabolic dysregulation, rather than just one, may have better clinical and systemic effects.

Peroxisome proliferator-activated receptors (PPARs) are ligand-activated nuclear receptors from the nuclear hormone receptor superfamily that act as heterodimerization partners with the retinoid X receptor (RXR) to regulate transcription of target genes with PPAR-responsive elements in their promoter regions [23,24]. Peroxisome proliferator-activated receptor alpha (PPARα), peroxisome proliferator-activated receptor beta/delta (PPARβ/δ), and peroxisome proliferator-activated receptor gamma (PPARγ) are expressed in various tissues, including the epidermis, dermis, sebaceous glands, and adipose tissue [25-27]. These receptors act as metabolic signal integrators, with endogenous ligands such as fatty acids, eicosanoids, and other lipid metabolites produced during inflammation and wound repair [28,29]. PPARs play a role in metabolic regulation, including glucose homeostasis, fatty acid oxidation, and adipogenesis. They also have anti-inflammatory and immunomodulatory functions by suppressing pro-inflammatory transcription factors such as nuclear factor κB (NF-κB), signal transducer and activator of transcription 3 (STAT3), and activator protein (AP)-1 [30-32].

The hypothesis that PPAR dysfunction is important to psoriasis pathogenesis came from converging observations: (1) genetic connections between PPAR polymorphisms and psoriasis susceptibility [33]; (2) PPARγ and PPARα are consistently downregulated in psoriasis lesions and animal models [34-36]; (3) upregulation of PPARβ/δ in psoriatic epidermis, especially in suprabasal differentiated keratinocytes [37,38]; and (4) therapeutic responsiveness of preclinical psoriasis models to PPAR agonists [38-41]. These findings lend support to a disease model in which PPAR isoform dysregulation connects metabolic dysfunction and T-cell-mediated inflammation, potentially explaining why psoriasis patients with metabolic comorbidities have more severe, treatment-resistant symptoms.

**Figure 1:**
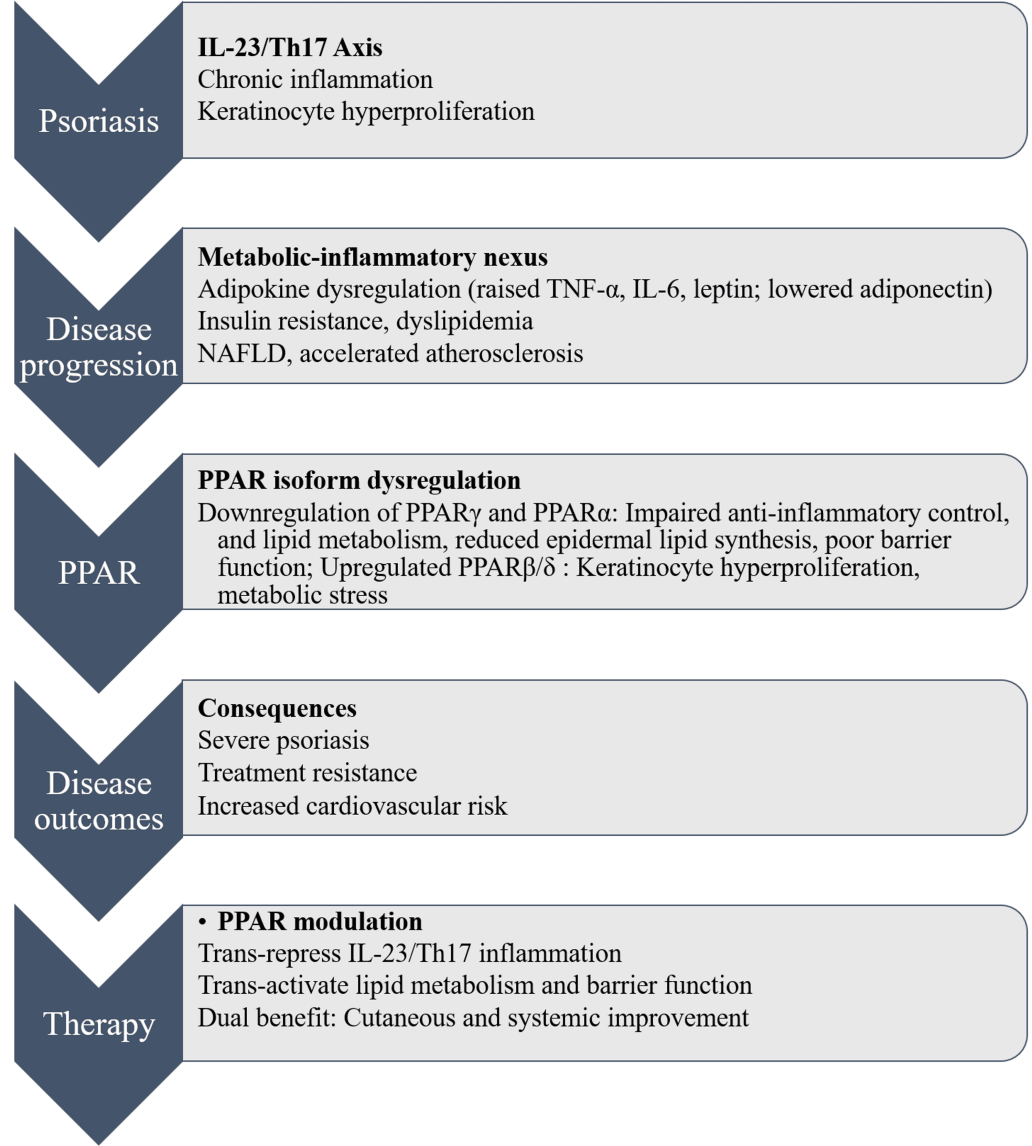
Mechanistic insights into psoriasis disease progression. This image has been synthesized from references [2,11,20,21,25-27,37-41]. IL-23: interleukin-23; Th17: thymus lymphocyte helper component 17; TNF-α: tumor necrosis factor-alpha; IL-6: interleukin-6; NAFLD: non-alcoholic fatty liver disease; PPAR: peroxisome proliferator-activated receptor; PPARγ: peroxisome proliferator-activated receptor gamma; PPARα: peroxisome proliferator-activated receptor alpha; PPARβ/δ: peroxisome proliferator-activated receptor beta/delta

According to this review, PPAR signaling modulation provides a dual-benefit approach that simultaneously reduces cutaneous inflammation by trans-repressing the IL-23/Th17 axis and trans-activating genes that regulate lipid metabolism, barrier function, and metabolic homeostasis. This approach addresses both the skin lesion and the underlying systemic disturbance that characterizes the *psoriatic march*. The difficulty, however, is not limited to finding PPAR-active substances; it also includes isoform-specific selectivity, cellular context, temporal dynamics of activation, and pragmatic concerns about bioavailability and side effects (Figure 1).

To realize the therapeutic potential of PPAR-directed therapy in psoriasis and systemic consequences, this review identifies important translational gaps, summarizes current mechanistic and clinical evidence, and suggests a research plan.

## Review

PPAR isoform landscape in psoriasis

PPAR-γ: Anti-inflammatory Anchor and Downregulation in Skin Lesions

PPARγ is mostly expressed in differentiated suprabasal keratinocytes and plays a vital role in maintaining inflammatory tolerance and epidermal homeostasis [42]. A 2021 analysis found a ~5-fold reduction in lesional versus nonlesional epidermis, and quantitative polymerase chain reaction (PCR) in patient biopsies (n = 23) confirmed this deficit, which was partially restored after laser phototherapy [36]. These seminal studies consistently show significant downregulation of PPARγ messenger RNA (mRNA) and protein in psoriatic skin lesions compared to non-lesional or healthy control skin. Subtle but persistent PPARγ downregulation in lesions was confirmed by microarray analysis [43]. Because PPARγ is typically prevalent in the superficial dermis and stratum granulosum, where it preserves barrier integrity and inhibits innate immune activation, this loss is significant.

Upstream regulators of PPARγ expression suppression include NF-κB, STAT3, AP-1, fos-like antigen 1 (FOSL1), aryl hydrocarbon receptor (AHR), GATA binding protein 3 (GATA3), hypoxia-inducible factor 1-alpha (HIF1A), and forkhead box O1 (FOXO1), all of which are hyperactivated in psoriatic inflammation [44, 45]. Pattern recognition receptors, including epidermal growth factor (EGFR), and metabolic sensors such as mechanistic target of rapamycin (mTOR), 5' adenosine monophosphate-activated protein kinase (AMPK), phosphorylate and suppress PPARγ transcription or cause ubiquitination [46]. Cytokines associated with psoriasis, such as TNF-α and IL-1β, upregulate negative PPARγ regulators while downregulating the receptor itself through post-translational changes [47].

Molecular mechanisms: trans-repression of NF-κB and STAT3

PPARγ’s anti-inflammatory effect stems from its capacity to block pro-inflammatory transcription factors through protein-protein interactions and functional antagonism, rather than directly activating genes (trans-activation) [48,49]. PPARγ undergoes conformational changes and recruits coactivators, such as steroid receptor coactivator 1 (SRC1) and CREB-binding protein (CBP/p300), to bind to PPAR-responsive elements upon ligand binding (natural agonists: 15d-prostaglandin J2, 9-hydroxyoctadecadienoic acid (9-HODE), palmitoleic acid; synthetic agonists: thiazolidinediones pioglitazone and rosiglitazone; natural ligands: curcumin, resveratrol). To bind to PPAR-responsive elements in promoters of anti-inflammatory genes such as IL-10, FOXP3, and inhibitor of kappa-light-chain-enhancer of activated B cells alpha (IκBα), PPARγ undergoes conformational change and attracts coactivators (steroid receptor coactivator 1 (SRC1), CREB-binding protein (CBP/p300) [50,51]. Crucially, the p65 and p50 subunits of NF-κB and the DNA-binding domain of STAT3 are physically interacted with by activated PPARγ heterodimer (PPARγ/RXR), which sequesters them from chromatin and stops them from trans-activating pro-inflammatory genes for IL-17, IL-23, TNF, IL-6, IL-1β, and S100A8/A9 [52,53].

This brake is released in psoriatic lesions, where low PPARγ levels allow constitutive activation and prolonged trans stimulation of the IL-23/Th17 module by failing to sequester NF-κB and STAT3. Primary human keratinocytes treated with TNF α/interferon gamma (IFN-γ) show increased IL-17A and IL-23 production in vitro, which is reversed by PPARγ agonists (pioglitazone, rosiglitazone, 15d-PGJ2) in a dose- and time-dependent manner [54,55]. Mechanistically, PPARγ agonism suppresses expression of retinoic acid-related orphan receptor gamma t (RORγt), the master transcription factor driving Th17 differentiation, inhibits STAT3 phosphorylation at Y705 (preventing its nuclear accumulation and DNA binding), and decreases phosphorylation of nuclear factor of kappa light polypeptide gene enhancer in B-cell inhibitor alpha (IκBα), sequestering p65 in the cytoplasm [56,57].

Genetic models provide additional evidence: mice with keratinocyte-specific deletion of PPARγ develop spontaneous skin inflammation and are more susceptible to experimentally induced psoriasiform dermatitis (imiquimod), characterized by increased epidermal infiltration of Th17 cells, elevated IL-17 production, and expanded Langerhans cell populations [58,59]. Overexpressing constitutively active PPARγ in the skin or administering PPARγ agonists reduces Th17 responses and improves disease in murine psoriasis models (imiquimod, IL-23 injection) [60,61]. PPARγ is expressed in dendritic cells, macrophages, and Th17 cells, where agonism suppresses IL-23 production by dendritic cells and IL-17 secretion by Th17 cells, while promoting regulatory T cell (Treg) differentiation via induction of FOXP3 [62,63]. This latter mechanism is especially significant. PPARγ agonists decrease DNMTs and stabilize FOXP3 expression in naïve T cells, resulting in a suppressive immunological response [64].

STAT3 as a Central Node

STAT3 is a crucial hub connecting PPAR dysfunction to psoriatic disease, according to recent network and route analysis studies [36]. Activated by IL-23, IL-6, and TNF-α signaling, STAT3 concurrently (1) drives Th17 cell differentiation by trans-activating IL17, RAR-related orphan receptor C (RORC), and IL23R; (2) stimulates keratinocyte hyperproliferation by upregulating cyclin D1 and suppressing apoptotic genes; and (3) maintains the inflammatory loop by inducing IL-23 and TNF-α expression in dendritic cells and keratinocytes [65,66]. STAT3 phosphorylation is significantly increased in psoriatic lesions, and both immune cells and epidermal cells constitutively activate STAT3 [67]. About 36% of the genes in the IL-17/Th17 signaling pathway are repressed by PPARγ, according to network analysis of PPARγ targets. Multiple PPARγ-suppressed pro-inflammatory cascades, including NF-κB p65, Janus kinase 2 (JAK2), and mitogen-activated protein kinase (MAPK), are downstream of STAT3 phosphorylation/activation [36].

**Figure 2:**
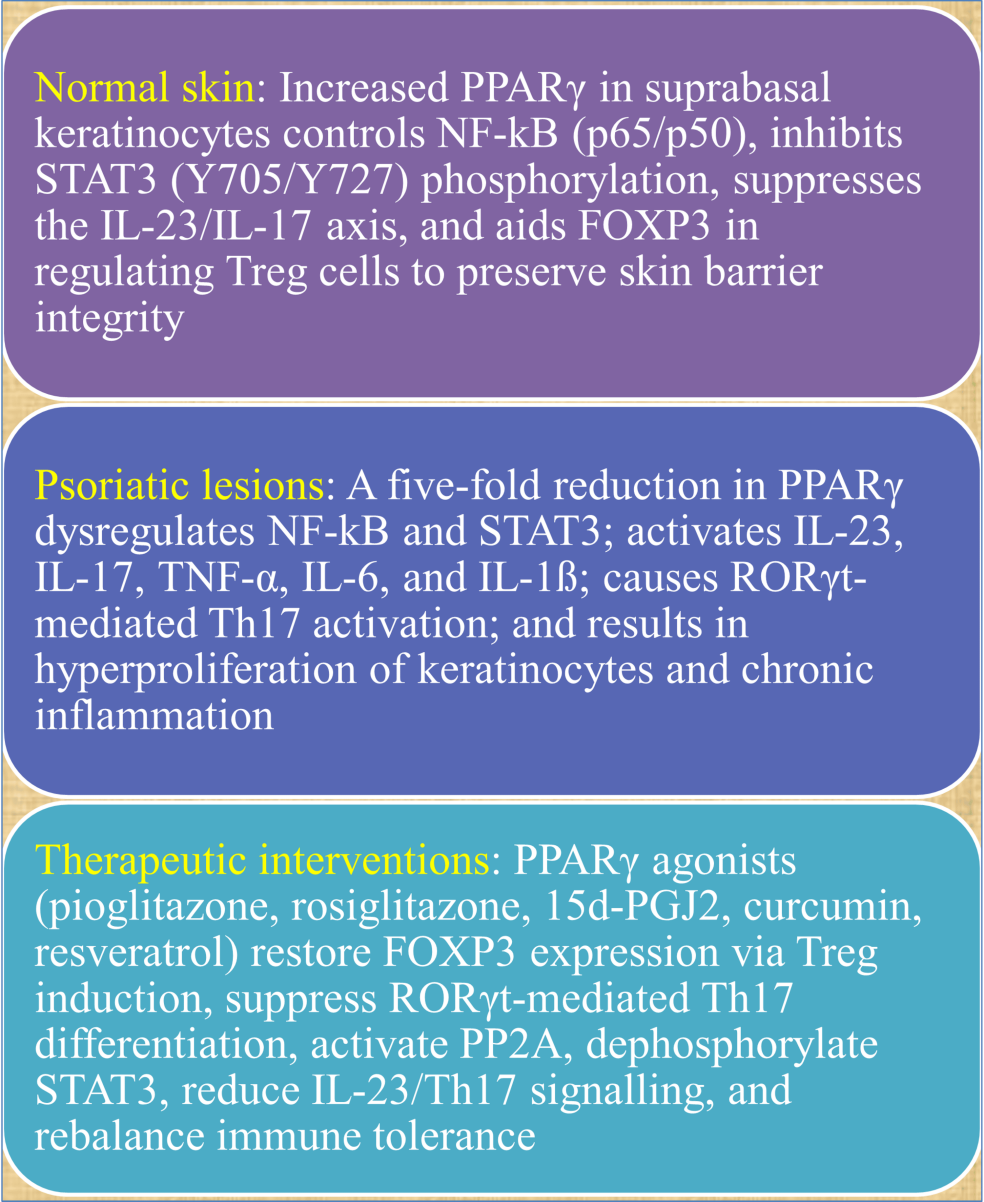
Mechanistic insights into PPARγ. This image has been synthesized from references [36,42,43,48-51,54,55,68-70]. NF-κB: nuclear factor κB; STAT3: signal transducer and activator of transcription 3; IL-23: interleukin-23; IL-17: interleukin-17; FOXP3: forkhead box P3; PPARγ: peroxisome proliferator-activated receptor gamma; TNF-α: tumor necrosis factor-alpha; IL-1β: Interleukin-1 beta; RORγt: retinoic acid-related orphan receptor gamma t; Th17: thymus lymphocyte helper component 17; 15d PGJ2: prostaglandin derived from arachidonic acid; PP2A: protein phosphatase 2A

Significantly, PPARγ agonism directly inhibits STAT3 phosphorylation at Y705 and Y727, which is mediated by increased protein phosphatase 2A (PP2A) activity [68,69]. This phosphatase-activating characteristic sets PPARγ apart from many immune-suppressive strategies and explains why PPARγ activation is particularly effective in reducing inflammation in a variety of STAT3-driven conditions, including inflammatory bowel disease (IBD), psoriasis, and rheumatoid arthritis (RA) [70] (Figure 2).

PPAR-α: Unexplored Barrier Guardian and Its Role in Epidermal Lipid Synthesis

The most prevalent PPAR isoform in muscle and liver, PPARα, is also expressed in the epidermis and has a specific function in barrier lipogenesis and the control of epidermal lipid composition [71,72]. The main barrier to transepidermal water loss and pathogen penetration is the epidermal permeability barrier, which is found in the stratum corneum and is made up of three lipid classes, namely, ceramides (50%), fatty acids (25%), and cholesterol (25%), arranged into lamellar sheets [73]. Nuclear receptors such as PPARα, liver X receptors (LXRs), vitamin D receptors (VDRs), and others control the production and secretion of very-long-chain (C24/C26) ceramides and saturated fatty acids, which are essential for maintaining normal barrier homeostasis [74,75].

High‑throughput transcriptome analysis of clinical psoriasis identified over 1,000 differentially expressed genes, including ≈300 upregulated inflammatory mediators and ≈200 downregulated regulatory pathways. These findings highlight keratinocytes’ active role in amplifying immune responses and metabolic dysfunction, reinforcing psoriasis as a systemic inflammatory disease beyond skin manifestations [76]. Analysis of lipid metabolism genes in psoriasis reveals suppression of PPARα-target genes involved in fatty acid activation of acyl-CoA synthetase long-chain family members (ACSL1-6), carnitine palmitoyltransferase 1 (CPT1), and β-oxidation, and there is a 5.58- to 7.58-fold decrease in PPARα mRNA in lesional versus nonlesional skin [77]. Additionally, 3-hydroxy-3-methylglutaryl-CoA synthase 1 (HMGCS1), 3-hydroxy-3-methylglutaryl-coenzyme A reductase (HMGCR), and serine palmitoyltransferase long-chain base subunit 1 (SPTLC1) were shown to be impacted in the cholesterol/ceramide biosynthesis [78,79]. As a result, psoriatic skin has quantitative and qualitative abnormalities in barrier lipids, including lower proportions of long-chain (C22-C26) ceramides, altered fatty acid chain lengths, and cholesterol depletion in the stratum corneum lipid matrix [80,81]. This lipid deficiency leads to increased transepidermal water loss (TEWL), decreased skin hydration, and poor barrier integrity, as seen in PPARα-knockout or PPARα-antagonist-treated mice [38,82].

Activation of Lipid Synthetic Pathways

PPARα heterodimerizes with RXR and binds PPAR-responsive elements in promoter regions of genes encoding enzymes essential for barrier lipogenesis and remodeling, such as sterol regulatory element-binding protein 1c (SREBP-1c), stearoyl-CoA desaturase 1 (SCD1), subunits of serine palmitoyltransferase (SPT)-SPTLC1 and SPTLC2, ceramide synthases (CERS), ACSL, and ATP-binding cassette transporters (ABCA12). Among these, SREBP-1c is a master regulator of lipogenic gene expression that stimulates the production of fatty acid synthase, acetyl-CoA carboxylase, and malonyl-CoA [83]. SCD1 modifies lipid fluidity by converting saturated to monounsaturated fatty acids [84]. De novo sphingolipid biosynthesis is catalyzed by SPTLC1/2 and CERS [85]. Fatty acids are activated by ACSL to be incorporated into complex lipids [86]. Lipids are packaged and exocytosed into the extracellular stratum corneum matrix with the help of ABCA12 [87]. These genes are upregulated by PPARα agonism in cultured human keratinocytes, which improves barrier permeability in reconstructed epidermal models, increases intracellular ceramide and fatty acid concentration, and enhances lamellar body formation [37,88]. Restoring PPARα signaling would theoretically restore barrier lipogenesis, lower TEWL, and attenuate irritant-induced and infection-triggered inflammatory cascades in the context of psoriasis, where barrier failure is both a cause and an effect of inflammation.

Clinical and translational status

**Figure 3:**
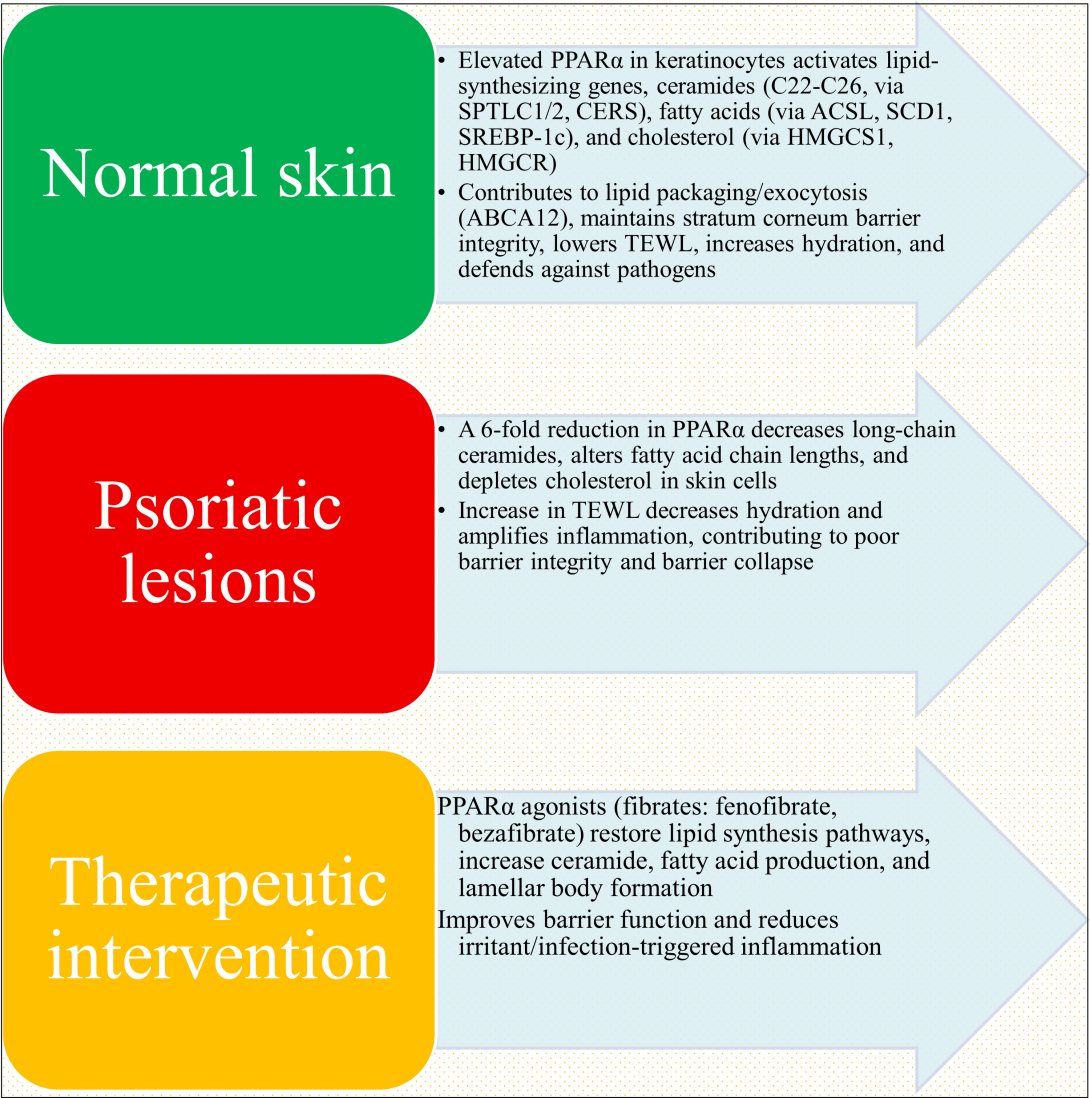
Mechanistic insights into PPARα. This image has been synthesized from references [37,38,71-75,77,88-90]. PPARα: peroxisome proliferator-activated receptor alpha; SPTLC1/2: serine palmitoyltransferase long chain base subunit 1/2; CERS: ceramide synthase; ACSL: acyl-coA synthetase; SCD1: stearoyl-coA desaturase 1; SREBP-1c: sterol regulatory element-binding protein-1c; HMGCS1: 3-hydroxy-3-methylglutaryl-CoA synthase 1; HMGCR: 3-hydroxy-3-methylglutaryl-coenzyme A reductase; ABCA 12: ATP-binding cassette subfamily A member 12; TEWL: transepidermal water loss

Fibrate (PPARα) agonists have surprisingly little direct clinical evaluation in psoriasis. Patients with psoriasis associated with dyslipidemia treated with fenofibrate or bezafibrate may see minor improvements in the psoriasis area and severity index (PASI) (20-35% reduction) in a few small observational studies and case reports [89,90]. However, these studies lack rigorous follow-up and placebo controls. The historical emphasis on PPARγ agonism for dermatological applications and the lesser anti-inflammatory effectiveness of PPARα relative to PPARγ in T-cell-mediated inflammation are probably the reasons for the paucity of fibrate research in psoriasis. However, studies combining fibrates with topical corticosteroids or calcineurin inhibitors show improved outcomes compared to monotherapy in animal models [37]. This suggests that dual modulation of inflammation (PPARγ) and barrier (PPARα) might yield superior therapeutic benefit, representing a significant translational gap that merits further investigation (Figure 3).

Debate Between Wound Healing and Hyperproliferation: PPAR-β/δ Overexpression and Contradictory Roles

PPARβ/δ is markedly increased in psoriatic lesional epidermis, in sharp contrast to the downregulation of PPARγ and PPARα [38,39]. All epidermal layers exhibit upregulation of PPARδ mRNA and protein, with accumulation being most noticeable in the granular and suprabasal keratinocytes of psoriatic plaques [38]. Subcellularly, PPARδ protein is primarily nuclear in granular cells but localizes to both the cytoplasm and the nucleus in basal and spinous layers, indicating constitutive activation by endogenous ligands in differentiated keratinocytes [38]. A feedforward loop that sustains PPARδ activation is created by co-upregulating fatty acid-binding protein 5 (FABP5), which carries endogenous PPARδ ligands to the nucleus, tracks PPARδ expression levels, and is a PPARδ target gene [38].

It is still debatable and paradoxical how increased PPARδ contributes to psoriasis. Depending on cellular environment, ligand concentration, and temporal dynamics, evidence suggests both potentially reparative (wound repair, differentiation) and pathogenic (hyperproliferation, Th17 amplification) activities. This debate highlights PPARδ’s dual role in tissue repair: during physiological wound healing, PPARδ activation suppresses apoptosis and increases keratinocyte migration, survival, and epithelialization through phosphoinositide 3-kinase (PI3K)/protein kinase B (Akt) signaling [91]. However, persistent PPARδ activation seems to be harmful in chronic inflammatory psoriasis.

Hyperproliferation Mechanism: Heparin-Binding Epidermal Growth Factor-Like Growth Factor and STAT3 Axis

Elevated heparin-binding epidermal growth factor (EGF)-like growth factor (HB-EGF), which stimulates EGFR and maintains the proliferative phenotype, contributes to keratinocyte hyperproliferation in psoriatic lesions [38,39,92]. PPARδ is a direct transcriptional activator of HB-EGF: PPARδ agonists (GW501516, GW0742, L-165041) increase HB-EGF mRNA and protein levels in human keratinocytes, boosting EGFR-mediated proliferation [93]. On the other hand, PPARδ antagonism or genetic deletion (e.g., GSK0660, GSK3787) suppresses proliferation by lowering HB-EGF expression [38]. Crucially, transgenic mice exhibit a psoriasis-like phenotype with epidermal hyperplasia, enhanced Th17 infiltration, and persistent STAT3 activation after suprabasal overexpression of constitutively active human PPARδ and topical treatment of PPARδ agonist (GW501516) [38]. Subsequent therapy with a PPARδ antagonist reverses this phenotype, suggesting that PPARδ is causally harmful in this paradigm.

A feedforward inflammatory loop is involved in the process: activation of suprabasal PPARδ IL-22 stimulates terminal keratinocyte differentiation and concurrently triggers the production of pro-inflammatory cytokines, specifically IL-1β, IL-36, IL-17, and IL-23, through a mechanism that is mostly dependent on STAT3. These cytokines stimulate the production of IL-23, which polarizes naïve T cells toward a Th17 phenotype by activating cutaneous dendritic cells. Simultaneously, the autocrine action of IL-1β on basal keratinocytes promotes proliferation and amplifies the HB-EGF signal. Psoriasis is characterized by a self-sustaining cycle of itching, scratching, and inflammation [38].

Metabolic Reprogramming: Glycolysis and Lipid Depletion

The metabolic reprogramming of keratinocytes toward anaerobic and away from oxidative phosphorylation (OXPHOS) is a novel and important mechanism by which PPARδ contributes to psoriatic pathogenesis. Keratinocytes in human psoriatic epidermis and lesional skin of mouse models (imiquimod-induced, flaky tail) have the following characteristics. Pyruvate dehydrogenase kinase 1 (PDK1), a PPARδ target gene that phosphorylates and deactivates pyruvate dehydrogenase (PDH), the rate-limiting enzyme linking glycolysis to mitochondrial oxidation, is upregulated in the stratum corneum, resulting in increased lactate production and accumulation [38]. This metabolic change, known as the Warburg effect, is energetically effective for maintaining fast cell division and reducing the generation of reactive oxygen species (ROS) in the mitochondria.

Acyl-CoA oxidase 1 (ACOX1) and other PPARδ-target genes are upregulated in the peroxisomal pathway, indicating increased peroxisomal fatty acid β-oxidation [38]. ACOX1 overexpression in psoriatic epidermis selectively targets C24 and C26 ceramides and fatty acids, the very lipids necessary for barrier function, and peroxisomes only oxidize very-long-chain (C>20) fatty acids [38]. In line with ACOX1 overexpression, lesional skin exhibits significantly lower proportions of barrier-critical very-long-chain ceramides [39]. This metabolic shift toward barrier-structural lipid β oxidation is an example of how PPARδ-mediated metabolic reprogramming directly damages the integrity of the skin barrier while promoting proliferation.

The functional outcome is striking: prolonged PPARδ activation consumes the very lipids needed for barrier repair while simultaneously promoting keratinocyte hyperproliferation and inflammation. This mechanistic paradox may account for the failure of simple PPARδ agonism as a treatment for psoriasis and the emergence of PPARδ antagonism as the more logical course of action.

GSK3787 and the Antagonism Strategy

Research on GSK3787, a selective, irreversible covalent antagonist of PPARβ/δ, provides crucial evidence for PPARδ antagonism [39]. Topical GSK3787 therapy lowers skin thickness, erythema, and epidermal infiltration of immune cells by 60-70% when compared to vehicle in mice with imiquimod-induced psoriasiform dermatitis [39]. GSK3787 inhibits the molecular expression of genes linked to psoriasis, such as IL-17, IL-23A, IL-22, IL-1β, and associated inflammatory mediators [39]. Mechanistically, antagonism creates a net anti-inflammatory impact by blocking PPARδ from trans-repressing anti-inflammatory transcription factors, such as B-cell lymphoma 6 (BCL-6), and from trans-activating pro-proliferative and pro-inflammatory genes [39]. Notably, GSK3787 has a better therapeutic profile for PPARδ antagonism as it does not cause the weight gain or metabolic adverse effects linked to PPARγ agonism.

Conflicting evidence and context-dependency

Nevertheless, some research indicates that PPARδ agonists (e.g., GW501516, GW0742) decrease experimental autoimmune encephalomyelitis and colitis in mice and lower pro-inflammatory cytokine production in macrophages [93,94]. This seeming paradox probably relates to ligand dose and cell-type selectivity, which are covered below.

**Figure 4:**
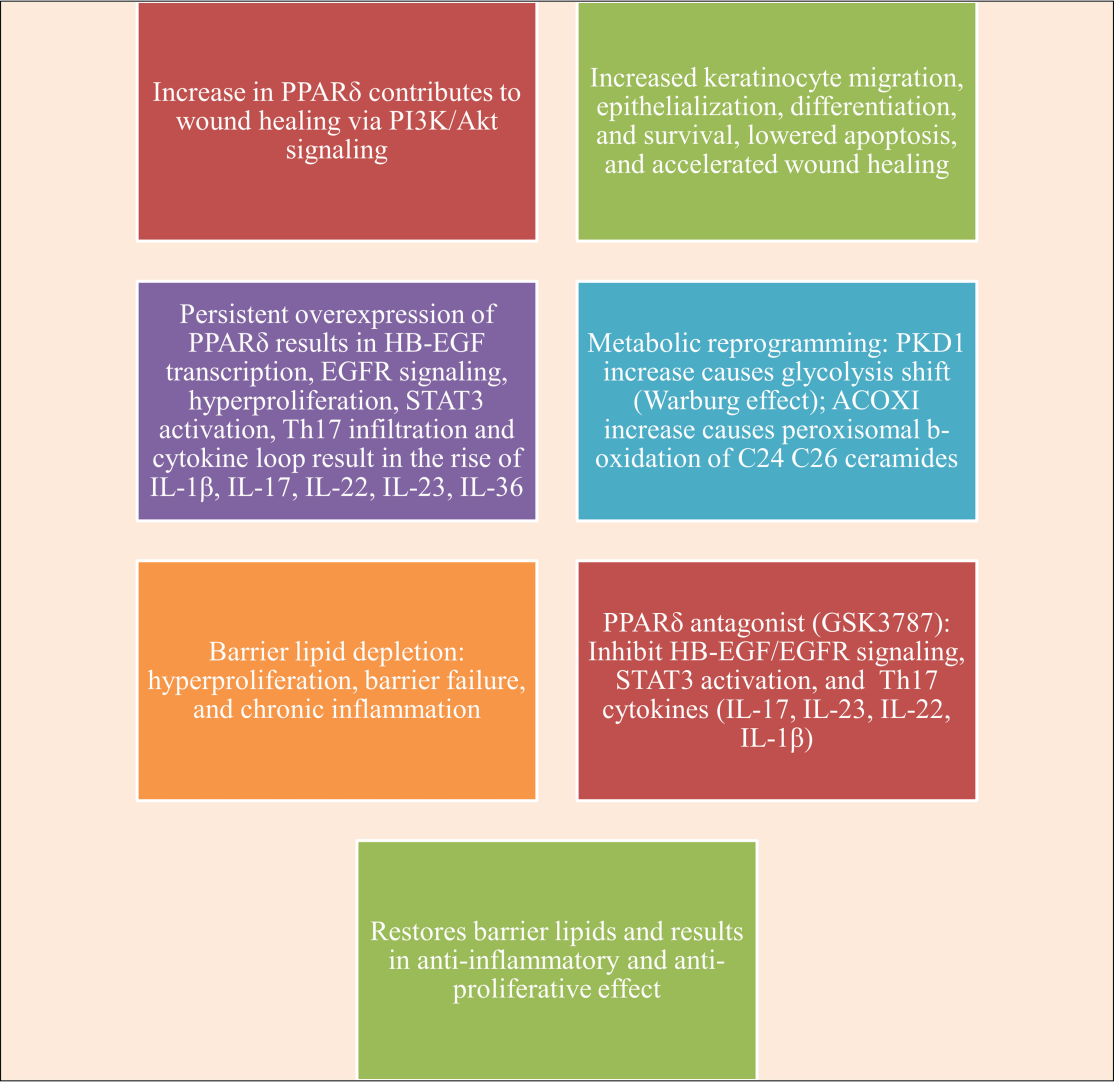
Mechanistic insights into PPARδ. This image has been synthesized from references [38,39,91-94]. PPARδ: peroxisome proliferator-activated receptor delta; PI3K/Akt: phosphoinositide 3-kinase/protein kinase B; HB-EGF: heparin-binding-epidermal growth factor; STAT3: signal transducer and activator of transcription 3; Th17: thymus lymphocyte helper component 17; IL-1β: interleukin-1 beta; IL-23: interleukin-23; IL-17: interleukin-17; IL-22: interleukin-22; IL-36: interleukin-36; PKD1: pyruvate dehydrogenase kinase 1; ACOX1: acyl-CoA oxidase 1; EGFR: epidermal growth factor receptor; GSK3787: 4-chloro-N-(2-{[5-trifluoromethyl)-2-pyridyl]sulfonyl}ethyl)benzamide

PPARδ agonism in immune cells may decrease NF-κB-mediated inflammatory gene expression and encourage differentiation toward non-inflammatory phenotypes at physiological doses. Unbalanced trans-activation of proliferative/inflammatory genes overprotective trans-repression of anti-inflammatory factors causes PPARδ to become pathogenic in epidermal cells at supraphysiological ligand doses or in the setting of persistent overexpression (transgenic models). Acute agonism and persistent constitutive activation may result in different kinetics of ligand binding and the recruitment of different coactivator/repressor complexes. A crucial translational problem is highlighted by this context-dependency and dose-response complexity: basic PPAR agonism is an inadequate psoriasis treatment approach and may even be inappropriate for PPARδ. On the other hand, specific PPARδ antagonism, as opposed to wide agonism, emerges as a potential approach that needs clinical development (Figure 4).

Psoriatic comorbidome, metabolic syndrome, and the systemic bridge: the IL-23/Th17 axis and systemic symptoms

Although crucial for cutaneous inflammation, the IL-23/Th17 axis is not exclusive to the skin. There is growing evidence that psoriatic skin illness spreads these signals systemically, causing distant tissue inflammation and cardiometabolic dysfunction [6,95]. IL-23 and Th17 cells are systemic drivers of autoimmune and inflammatory diseases. In psoriasis patients, elevated levels of TNF-α, IL-6, IL-17A, and IL-23 are correlated with both systemic comorbidities and the severity of the illness [96]. These cytokines cause endothelial dysfunction and vascular inflammation: TNF-α and IL-17 stimulate endothelial cells to produce intracellular adhesion molecules (ICAM-1) and vascular cell adhesion molecules (VCAM-1), as well as to attract neutrophils and monocytes to the vasculature, hastening the development and destabilization of atherosclerotic plaque [11,95]. Metabolic dysregulation: TNF-α and IL-6 increase the synthesis of IL-23 in the liver, decrease the anti-inflammatory adipokine adiponectin, and increase leptin and other pro-inflammatory adipokines. These signals together disrupt insulin transmission and increase visceral adiposity [20, 97]. Joint inflammation: IL-23 and Th17 cells cause enthesitis and synovitis in prostate-specific antigen (PsA) by recruiting neutrophils and osteoclast-activating cells and producing IL-17A and IL-22 locally [98-100].

**Figure 5:**
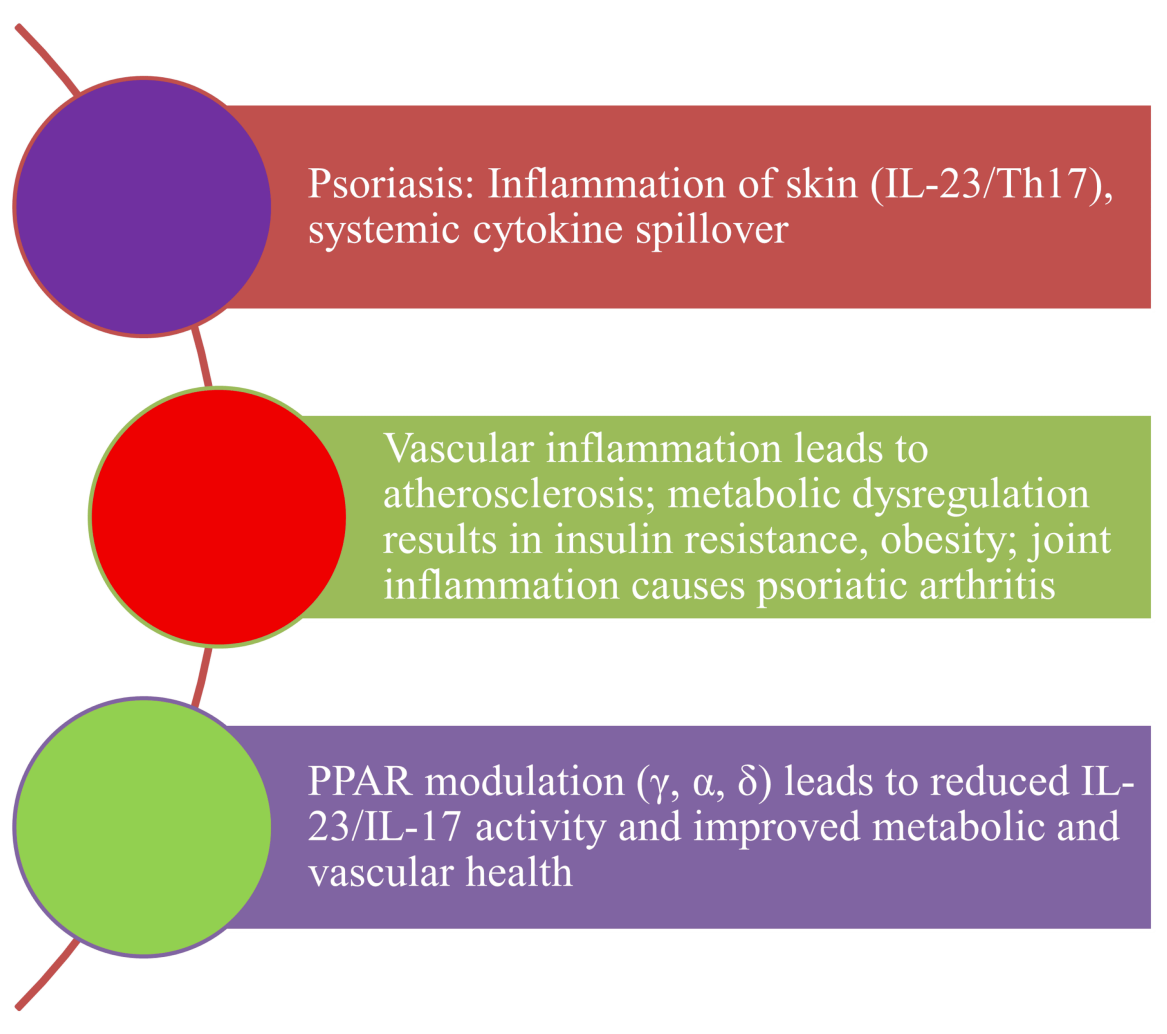
The psoriatic comorbidome cycle. This image has been synthesized from references [6,11,20,95-100]. IL-23: interleukin-23; Th17: thymus lymphocyte helper component 17; PPAR: peroxisome proliferator-activated receptor

Finding treatment targets that address both cutaneous and systemic IL-23/Th17 activity is motivated by the systemic spread of psoriatic inflammation. Here, PPARs may work in concert: PPAR agonism (especially PPARγ) modulates the differentiation and function of systemically distributed immune populations (dendritic cells, macrophages, Th17 cells, Tregs) and enhances metabolic parameters that subsequently decrease pro-inflammatory signaling, in addition to locally suppressing keratinocyte IL-23/IL-17 production (Figure 5).

Metabolic dysfunction: obesity, insulin resistance, NAFLD, and lipid dysregulation

The Vicious Cycle: Psoriasis ↔ MeS

Epidemiological research indicates a significant association between moderate-to-severe psoriasis and MeS, obesity, type 2 diabetes mellitus (T2DM), dyslipidemia, and NAFLD that goes beyond what is expected by conventional cardiovascular risk factors alone [3,4,101]. The link is bidirectional and may be causative. Obesity and metabolic dysfunction increase the incidence and severity of psoriasis (obesity is an independent psoriasis risk factor in genome-wide association studies) [102], while psoriatic inflammation aggravates metabolic dysregulation through systemic cytokine signaling [103,104]. The *psoriatic march* is a self-sustaining cycle that begins with cutaneous psoriasis and advances to cardiovascular disease, MeS, and, if untreated, early death [2].

Adipokine dysregulation and malfunction of adipose tissue are the mechanisms behind this reciprocal interaction [20]. Expanded visceral adipose tissue becomes chronically inflamed in obesity due to increased M1 (pro-inflammatory) macrophage infiltration, increased production of TNF-α, IL-6, monocyte chemoattractant protein-1 (MCP-1), and leptin, and decreased production of anti-inflammatory adipokines (adiponectin, IL-10) [105]. These inflammatory signals originating from adipose tissue systemically stimulate Th17 differentiation, inhibit Treg formation, increase IL-23 production in dendritic cells, and disrupt insulin signaling in peripheral organs (liver, muscle).

Simultaneously, hepatic lipid accumulation (NAFLD) and obesity-associated dyslipidemia (high triglycerides, low HDL) increase systemic inflammation by activating pattern recognition receptors such as Toll-like receptors (TLRs) and NOD-like receptors (NLRs) by lipid metabolites and free fatty acids [106,107]. This obesity-inflammatory load reduces the threshold for disease flare-ups in people who already have psoriasis and encourages the development of systemic symptoms.

Role of PPARs in Metabolic Homeostasis

The pathophysiology of MeS is closely associated with the malfunctioning of PPARs, which are canonical metabolic sensors. Insulin sensitivity is enhanced by PPARγ agonism via several mechanisms: (1) improvement of insulin-stimulated glucose uptake in muscle through the translocation of glucose transporters (GLUT4) to the cell membrane [108]; (2) stimulation of adipocyte production of adiponectin, an anti-inflammatory adipokine that increases insulin signaling and suppresses hepatic glucose production [37,109]; and (3) suppression of TNF-α and IL-6 from immune cells and adipose tissue, which improves hepatic insulin signaling and reduces chronic systemic inflammation [32,33].

Similarly, PPARα agonism (fibrates) stimulates lipid catabolism by upregulating genes that encode fatty acid β-oxidation enzymes (CPT1, ACSL, and AMPK), suppressing very-low-density lipoprotein (VLDL) synthesis by lowering hepatic triglyceride concentration, and increasing high-density lipoprotein (HDL) production [110,111]. In skeletal muscle, PPARδ/β agonism increases oxidative metabolism and encourages the transition from glycolysis to fatty acid oxidation, which improves metabolic flexibility and insulin sensitivity [112,113]. On the other hand, metabolic capacity is compromised in psoriasis, where all three PPAR isoforms are dysregulated (PPARγ and PPARα downregulated; PPARδ upregulated). This is because loss of PPARγ reduces adiponectin production and permits unrestrained TNF-α/IL-6 secretion, loss of PPARα compromises fatty acid and lipid metabolism, and pathological PPARδ upregulation in immune cells and hypometabolic keratinocytes drives glycolysis instead of oxidative metabolic syndrome, even in non-obese psoriasis patients [38].

NAFLD as a Disease Comorbidity

NAFLD, which affects 30-60% of psoriasis patients compared to about 25% of the general population, is a particularly remarkable junction of MeS and psoriasis [114]. NAFLD, which includes cirrhosis, nonalcoholic steatohepatitis (NASH), and simple steatosis (NAFL), is an independent predictor of metabolic problems and cardiovascular events. Genes controlling inflammation and lipid metabolism, such as patatin-like phospholipase domain-containing protein 3 (PNPLA3), lysophospholipase-like 1 (LYPLAL1), and transmembrane 6 superfamily member 2 (TM6SF2), have overlapping loci for psoriasis and NAFLD, according to genome-wide association studies, indicating a common genetic predisposition [115]. Elevated TNF-α in psoriasis promotes hepatic de novo lipogenesis and inhibits fatty acid oxidation, favoring triglyceride accumulation, which is indicative of TNF-α-mediated hepatic lipid accumulation [2]. Similar to skin, psoriasis-related TNF-α and IL-6 inhibit the expression of PPARα and PPARγ in the liver, limiting their anti-inflammatory and metabolic-regulating roles in hepatocytes and Kupffer cells (liver macrophages), hence confirming PPAR dysregulation in the liver [105,116].

**Figure 6:**
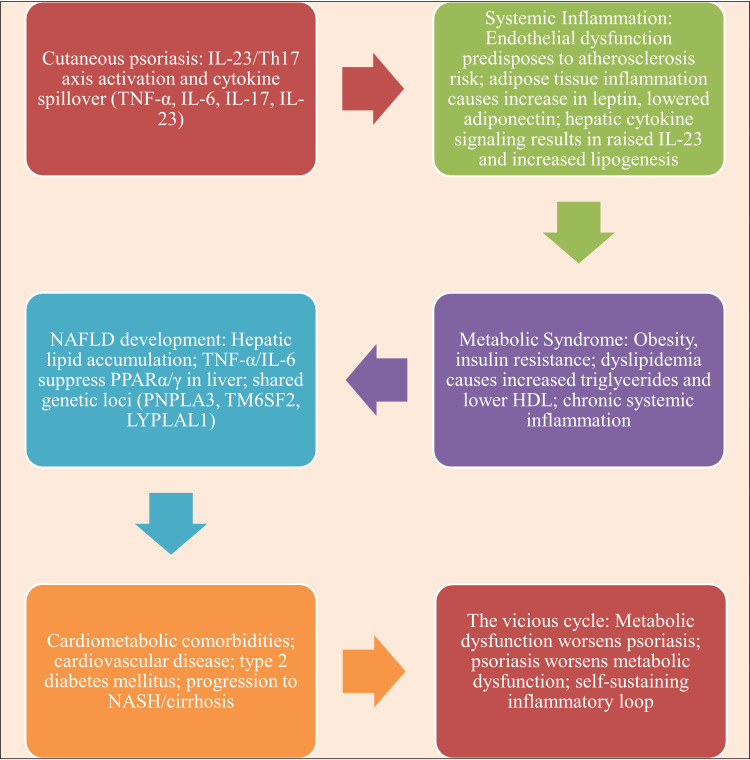
The psoriatic march flow. This image has been synthesized from references [2-4,20,32,33,37,38,101-118]. IL-23: interleukin-23; Th17: thymus lymphocyte helper component 17; TNF-α: tumor necrosis factor-alpha; IL-9: interleukin-6; IL-17: interleukin-17; HDL: high-density lipoproteins; NAFLD: non-alcoholic fatty liver disease; PPARα/γ: peroxisome proliferator-activated receptor alpha/gamma; PNPLA3: patatin-like phospholipase domain-containing protein 3; TM6SF2: transmembrane 6 superfamily member 2; LYPLAL1: lysophospholipase-like 1; NASH: non-alcoholic steatohepatitis

Therefore, PPARγ agonism (pioglitazone) improves NAFLD in the context of psoriasis, as shown in small randomized controlled trials and observational cohorts where pioglitazone improved NAFLD histological features and decreased hepatic triglyceride content in patients with psoriasis and concurrent metabolic dysfunction [117,118]. The justification for PPAR-directed therapy as a *metabolic checkpoint* intervention is further supported by this dual benefit (hepatic and cutaneous improvement) (Figure 6).

Cardiovascular and musculoskeletal comorbidities: role of PPARs beyond skin

Cardiovascular Disease Epidemiology and Pathophysiology: From Skin Inflammation to Atherosclerosis

Independent of conventional risk factors such as hypertension, diabetes, smoking, and dyslipidemia, patients with psoriasis had a 1.25-1.9-fold increased relative risk of CVD, coronary artery disease, myocardial infarction, stroke, and peripheral artery disease [5,6,95]. Across 31 observational studies with 1.6 million participants, psoriasis was linked to a 25-50% higher CVD risk. Severe cases showed three‑fold odds of myocardial infarction, ≈60% higher stroke risk, ≈40% higher cardiovascular death, and 21% increased all‑cause mortality, underscoring psoriasis as a systemic inflammatory condition with major long‑term health consequences [4]. Furthermore, compared to age- and risk factor-matched controls, psoriasis patients show signs of accelerated atherosclerosis, including higher coronary artery calcification scores, a higher burden of noncalcified (high-risk) plaques, and increased vascular inflammation (measured by 18F-fluorodeoxyglucose positron emission tomography) [2,6,119,120].

The systemic spread of TNF-driven and Th17-mediated inflammation is the molecular link between distant vascular illness and psoriatic skin inflammation [5]. Systemic activation of vascular endothelial cells, promotion of adhesion molecule expression (ICAM-1, VCAM-1, P-selectin), recruitment of pro-atherogenic immune cells (Th17 cells, neutrophils, inflammatory monocytes), enhancement of vascular smooth muscle cell proliferation, and acceleration of foam cell formation via uptake of oxidized LDL are all facilitated by elevated circulating IL-17A, TNF-α, and inflammatory cytokines from psoriatic lesions and expanded Th17 populations [11,121,122]. Additionally, through conventional metabolic-inflammatory pathways (TNF-α/IκB kinase, AMPK suppression, mTOR activation), psoriasis-related dyslipidemia, IR, and visceral obesity increase vascular inflammation [105].

PPAR Agonism and Vascular Protection

Beyond glycemic management, PPARγ agonists have proven cardioprotective benefits [32,33]. Anti-atherosclerotic effects: Pioglitazone lowers triglycerides, increases HDL, and decreases circulating low-density lipoprotein (LDL) cholesterol (especially small, dense, pro-atherogenic particles); it also lessens the pro-atherogenic response to LDL by inhibiting the expression of scavenger receptors on macrophages, which limits the formation of foam cells [37,110]. Anti-inflammatory vascular effects: PPARγ agonism reduces adhesion molecule expression, cytokine production (IL-6, TNF-α, IL-8), and the migration/proliferation of pro-atherogenic leukocytes by suppressing NF-κB and STAT3 activation in vascular endothelial cells and smooth muscle cells [111,123]. Additionally, PPARγ activation in endothelial cells increases the production of endothelial nitric oxide synthase (eNOS), which promotes anti-thrombotic signaling and vasodilation [124,125]. Anti-thrombotic effects: PPARγ agonists improve fibrinolytic function, inhibit procoagulant activity, and decrease platelet activation and aggregation [32, 33]. Myocardial protection: in models of myocarditis and heart failure, PPARγ agonism suppresses T-cell infiltration into the myocardium, improving cardiac function and reducing myocardial fibrosis through anti-inflammatory actions in cardiac myocytes and fibroblasts [87,110].

**Figure 7:**
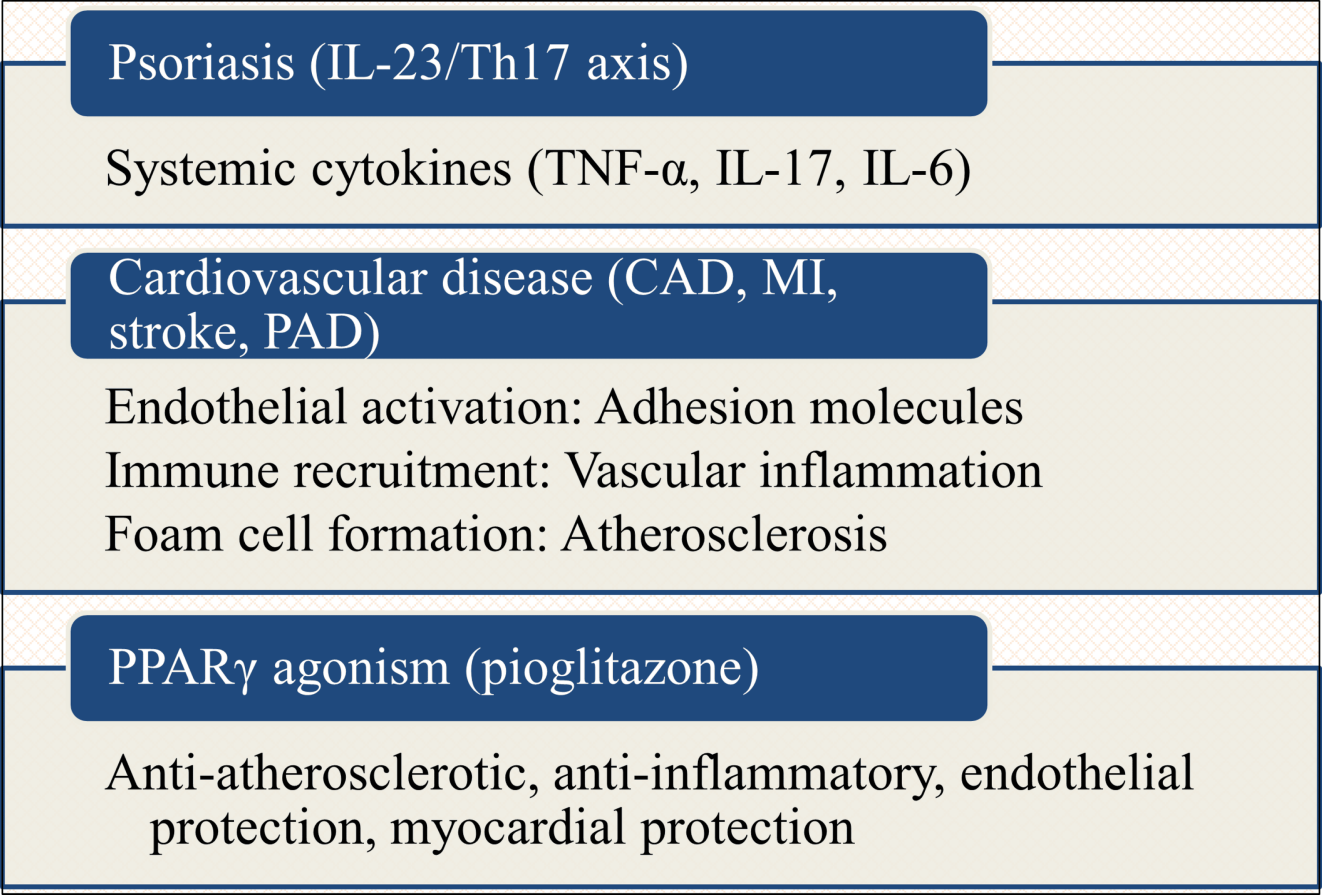
Psoriasis cardiovascular disease pathway. This image has been synthesized from references [2,4-6,11,32,33,87,95,105,111,119-125]. Th17: thymus lymphocyte helper component 17; IL-17: interleukin-17; TNF-α: tumor necrosis factor-alpha; IL-6: interleukin-6; CAD: coronary artery disease; MI: myocardial infarction; PAD: peripheral arterial disease; PPARγ: peroxisome proliferator-activated receptor gamma

Particularly for psoriasis patients, a combination of treatments that target metabolic factors (such as PPARγ agonism) and skin inflammation (such as biologics that target IL-17/IL-23) may reduce the risk of CVD. In fact, new observational data indicate that vascular inflammation (measured as decreased GlycA, a systemic inflammatory biomarker generated from glycan modifications of acute-phase proteins) is improved when psoriatic inflammation is controlled by biologics [6]. PPAR agonism may increase cardiovascular benefit through direct vascular actions, although definitive clinical trial confirmation is still pending (Figure 7).

Psoriatic arthritis: TNF-α, IL-17/IL-23, and systemic inflammation

Psoriatic Arthritis Pathogenesis and Overlap With Cutaneous Psoriasis

Psoriatic arthritis, found in approximately 30% of psoriasis patients, is a chronic inflammatory condition that affects the synovium, entheses (tendon/ligament insertion sites), and bone. It also causes cardiovascular and metabolic complications similar to cutaneous psoriasis [2,123]. The immunopathogenesis of psoriatic arthritis is similar to plaque psoriasis, with overexpression of TNF-α, IL-23, and IL-17 in synovium and entheseal tissue. Blocking agents targeting these cytokines (TNF-α inhibitors: adalimumab, infliximab, etanercept; IL-17 inhibitors: secukinumab, ixekizumab; IL-23 inhibitors: guselkumab, risankizumab) are highly effective in psoriatic arthritis [124]. PsA has more TNF-α-dependent pathophysiology than cutaneous psoriasis, with TNF-α playing a prominent role in joint inflammation and bone degradation [6].

Psoriatic arthritis is driven by TNF-α through both direct and indirect mechanisms. Directly, TNF-α promotes bone erosion by activating osteoclast precursors and mature osteoclasts via receptor activator of nuclear factor kappa-B ligand (RANKL)/receptor activator of nuclear factor kappa-B (RANK) signaling [125]. Indirectly, TNF-α increases the production of IL-23 by dendritic cells, which, in turn, promotes IL-6-mediated amplification of Th17 responses and expansion of joint-infiltrating neutrophils [126]. Furthermore, TNF-α encourages the migration of inflammatory monocytes to inflammatory joints, where they develop into fibroblast-like synovial cells (FLS) and pro-inflammatory macrophages, thereby sustaining the inflammatory cycle [127].

PPAR Modulation in Psoriatic Arthritis and Joint Inflammation

Although the role of PPARγ in psoriatic arthritis is less well understood than in cutaneous psoriasis, evidence from RA, a condition with significant mechanistic overlap with psoriatic arthritis, shows that PPARγ agonism reduces TNF-α and IL-6 production by FLS and macrophages, suppresses osteoclastogenesis, and reduces joint inflammation in mouse models of arthritis [32,33,37]. Osteoclast precursors, macrophages, T cells, and FLS in the inflammatory synovium express PPARγ; agonism inhibits the development of these pro-inflammatory cells while encouraging the growth of anti-inflammatory Tregs [33]. Additionally, PPARγ agonists reduce bone resorption and osteoclast development by suppressing the production of RANKL from immune cells and osteoblasts [32].

**Figure 8:**
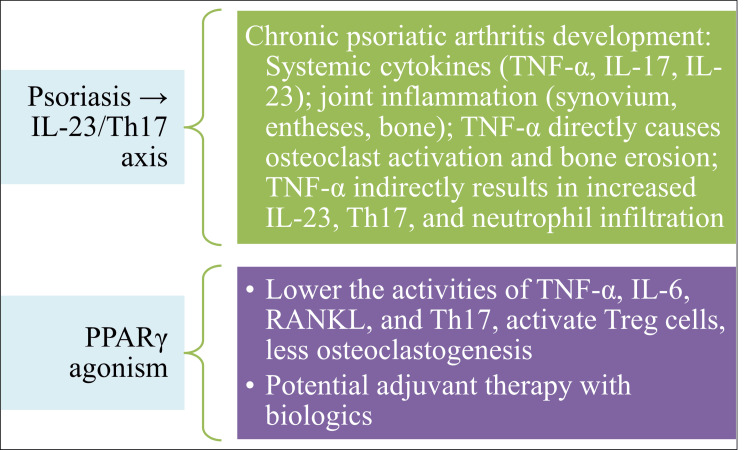
Pathogenesis of psoriatic arthritis. This image has been synthesized from references [2,6,32,33,37,110,124-127]. IL-23: interleukin-23; IL-17: interleukin-17; PPARγ: peroxisome proliferator-activated receptor gamma; TNF-α: tumor necrosis factor-alpha; IL-6: interleukin-6; Th17: thymus lymphocyte helper component 17; RANKL: receptor activator of nuclear factor kappa-B ligand; Treg: regulatory thymus lymphocytes

There are currently no large randomized controlled trials of PPARγ agonists in psoriatic arthritis; however, observational studies in RA patients with concurrent diabetes treated with pioglitazone report improvements in glycemic control and joint inflammation markers such as erythrocyte sedimentation (ESR) and C-reactive protein (CRP) [37,110]. These results imply that PPARγ agonism may be beneficial as an adjuvant for psoriatic arthritis, especially in individuals with metabolic comorbidities. Combination therapy that combines PPARγ agonism with TNF-α inhibitors or IL-17 inhibitors may be more effective than monotherapy due to psoriatic arthritis’s relative TNF reliance; this strategy is still awaiting clinical validation (Figure 8).

Therapeutic landscape: from clinical trials to future directions

Efficacy of Thiazolidinediones: PPAR-γ Agonists in Clinical Practice

The most researched TZDs for psoriasis are pioglitazone (Actos®, 15-45 mg/day) and rosiglitazone (Avandia®, 4-8 mg/day). Their effectiveness has been assessed in several randomized, placebo-controlled, or active-comparator studies.

Pioglitazone and Acitretin (2009)

Pioglitazone (30 mg/day) and acitretin (0.5-1.0 mg/kg/day) produced PASI-75 in 70% of participants in a 12-week randomized controlled study of 80 patients with moderate-to-severe plaque psoriasis, compared to 45% with acitretin alone and 20% with a placebo [128]. The combination showed synergy and was better than each agent alone. Hepatic enzyme increase (measured by alanine transaminase (ALT)/aspartate transaminase (AST) and acitretin’s teratogenicity were the primary concerns, with minimal side effects.

Pioglitazone Monotherapy (2014-2015)

In patients with mild plaque psoriasis, a double-blind randomized controlled trial (n = 52, 12-week duration) revealed that pioglitazone 15 mg daily produced PASI-50 in around 45% of patients compared to 12% in placebo, and PASI-75 in about 35% compared to placebo [129]. By week eight, erythema, scaling, and infiltration had all shown clinical improvement, indicating that PPAR modulation takes two to four weeks to have the best results. Significantly, metabolic indicators improved: fasting insulin improved, homeostasis model assessment of insulin resistance (HOMA-IR) dropped, and lipid profiles showed positive trends. There were reports of nausea and mild weight gain (1.5-2.0 kg).

Methotrexate and Pioglitazone (2019)

Standard treatment (methotrexate 7.5 mg/week), active treatment (pioglitazone 15 mg/day), and combination treatment (methotrexate + pioglitazone) were compared in a randomized, open-label, three-arm trial (n = 90, 12 weeks) [130]. According to the results, methotrexate alone showed a PASI-50 at week 12 in about 60% and PASI-75 in about 40%; pioglitazone alone showed PASI-50 in about 45% and PASI-75 in about 35%; and a combination therapy showed PASI-50 at week 8 in about 70%; at week 12, it was better than both monotherapies, with PASI-75 in about 60%. Additionally, the combination showed improved MeS measures (stable body weight, decreased HOMA-IR, improved lipid profile), indicating that concurrent metabolic treatment improved skin outcomes.

Rosiglitazone (2005-2010)

Early rosiglitazone open-label trials (n = 25) showed clinical improvements in erythema and induration and approximately 40% PASI-75 [131, 132]. Yet, because of worries about cardiovascular safety, pioglitazone has essentially replaced rosiglitazone; however, this debate has now been reexamined and partially disproved in more recent meta-analyses [133].

Meta-Analysis and Systematic Review Findings

A 2019 meta-analysis and systematic review of pioglitazone in psoriasis included seven randomized controlled trials (n = ~300-400 total across all studies) and came to the following conclusion about PASI-75 response: About 30-35% of patients with intermediate psoriasis attained PASI-75 with pioglitazone treatment, compared to 5-10% with a placebo, a relative risk improvement of about three to four times [134]. When paired with traditional medications (acitretin, methotrexate), pioglitazone produced PASI-75 in about 50-70% of cases, which was better than when either medication was used alone. Improved IR (HOMA-IR), fasting glucose, lipids, and inflammatory markers (CRP, TNF-α levels) were among the metabolic advantages of pioglitazone that were found to provide both systemic and cutaneous benefits. Weight increase (1.5-2.5 kg on average), gastrointestinal discomfort, infrequent fluid retention, and modest hepatic enzyme elevation (ALT/AST >3-fold upper limit of normal in ~3-5% of subjects; reversible following termination) were among the adverse events. There were no reports of opportunistic infections or severe hepatotoxicity.

Clinical significance and limitations

In patients with concurrent metabolic dysfunction (obesity, IR, dyslipidemia, NAFLD), pioglitazone has been shown to be a safe and somewhat effective add-on medication for moderate psoriasis. The limitations, however, included slow onset (8-12 weeks needed for maximal effect, compared to 2-4 weeks for most biologics); weight gain and metabolic side effects, including mild weight gain, may be problematic in patients with obesity-related psoriasis, potentially worsening the underlying metabolic dysfunction despite improved insulin sensitivity; and modest monotherapy efficacy (~30-35%) is inferior to that of modern biologic therapies (>80-90% PASI-75 rates with anti-IL-17, anti-IL-23, or anti-TNF-α agents).

Baseline and recurring liver function tests (ALT, AST, and bilirubin) are necessary for hepatotoxicity monitoring, which adds to the burden. Careful patient selection is required due to controversial long-term safety signals from large diabetes trials (PROactive, others) that raised concerns about increased fracture risk (especially in postmenopausal women) and increased bladder cancer risk (especially in men with long-term use >2 years) [135]. Large, multicenter, long-term randomized controlled studies in psoriasis are scarce, and the majority of the information comes from diabetes populations or tiny dermatology cohorts.

Pioglitazone is currently recommended for patients with mild plaque psoriasis coexisting with MeS, IR, or NAFLD who have exhausted traditional topical and phototherapy options and for whom biologic therapy is either contraindicated or rejected. It is not considered a first-line treatment for severe psoriasis or psoriatic arthritis.

Fibrates: PPAR-α agonists

Limited Clinical Evidence

Compared to thiazolidinediones, fibrates (bezafibrate, fenofibrate, and ciprofibrate) have comparatively few clinical trials in psoriasis. PASI improvements of 20-35% have been reported in a few small observational studies and case reports involving patients with psoriasis and dyslipidemia treated with fibrates; however, these studies lack placebo controls, standardized outcome measurements, and sufficient follow-up periods [88,136]. The recent literature did not find any published randomized controlled studies that explicitly evaluated fibrate monotherapy in psoriasis.

The moderate anti-inflammatory potency of PPARα is probably the reason for its limited clinical development. PPARα agonism significantly increases HDL and decreases triglycerides, but it trans-represses pro-inflammatory transcription factors (NF-κB, STAT3) less than PPARγ agonism, especially in T-cell-mediated settings [33]. Because of its wider anti-inflammatory profile and demonstrated effectiveness in autoimmune illnesses, PPARγ has been the focus of dermatological research throughout history. In contrast to the economic success of thiazolidinediones for diabetes, there is no pharmaceutical company investing in the development of fibrates as anti-psoriatic medicines.

Barrier-Fortifying Potential

Emerging preclinical evidence indicates that PPARα agonism may provide an additional benefit by restoring epidermal barrier function, despite the paucity of psoriasis-specific findings [38,137]. In murine atopic dermatitis models, combination studies revealed that fibrates improved barrier healing and decreased inflammation more than monotherapy when paired with anti-inflammatory drugs (topical corticosteroids, calcineurin inhibitors) [38]. Reexamining fibrate development for psoriasis, either as monotherapy in cases of mild disease or as adjunctive barrier-fortifying agents in combination regimens, is worthwhile because barrier dysfunction is both a cause and an effect of psoriasis, and because PPARα is downregulated in lesional skin. Pilot randomized controlled trials combining fibrates with topical anti-inflammatory medications or systemic biologics in individuals with concomitant dyslipidemia might be a practical strategy (Table 1).

**Table 1:** Comparative landscape of thiazolidinediones, fibrates, and biologics. This table has been synthesized from references [33,38,129-137]. TZDs: thiazolidinediones; PASI: psoriasis area and severity index; IL-23: interleukin-23; IL-17: interleukin-17; TNF-α: tumor necrosis factor-alpha; RCTs: randomized controlled trials; HOMA-IR: homeostasis model assessment of insulin resistance; CRP: C-reactive protein; HDL: high-density lipoprotein; TB: tuberculosis; CV: cardiovascular; IR: insulin resistance; NAFLD: non-alcoholic fatty liver disease

Feature	TZDs (pioglitazone, rosiglitazone)	Fibrates (fenofibrate, bezafibrate)	Biologics (IL-17, IL-23, TNF-α inhibitors)
Efficacy	PASI-75 ~30–35% (monotherapy); 50–70% in combination	PASI improvement ~20–35% (observational only; no RCTs)	PASI-75 >80–90%; PASI-90 ~70–80%; PASI-100 achievable in some
Onset of action	Slow (8–12 weeks)	Unknown; limited data	Rapid (2–4 weeks for maximal effect)
Metabolic benefits	Raised HOMA-IR, improved lipids, lowered CRP/TNF-α	Raised HDL, lowered triglycerides; modest anti-inflammatory	Neutral or mixed; some TNF-α inhibitors improve CV risk markers
Safety concerns	Weight gain, mild hepatic enzyme rise, fracture/bladder cancer risk (long-term, debated)	Limited psoriasis-specific safety data; generally safe in dyslipidemia	Immunosuppression risk (opportunistic infections, TB reactivation); injection-site reactions; rare malignancy signals
Barrier function	Limited evidence	Emerging preclinical support (epidermal repair)	Indirect improvement via reduced inflammation; not barrier-specific
Clinical development	Multiple RCTs, meta-analyses; moderate evidence base	Sparse; case reports, small observational studies	Extensive RCTs, long-term registries, real-world evidence
Best use case	Mild psoriasis with metabolic comorbidities (IR, NAFLD, obesity)	Psoriasis with dyslipidemia; potential adjunct for barrier repair	Moderate-to-severe psoriasis; psoriatic arthritis; first-line systemic therapy
Future potential	Combination with biologics; novel delivery systems; isoform-selective modulation	Adjunctive barrier repair; pilot RCTs needed	Personalized biologic sequencing; biosimilars; combination with small molecules

Novel delivery systems: overcoming bioavailability barriers

Bioavailability Conundrum

Poor topical bioavailability is a crucial translational gap that limits PPAR ligand potency in psoriasis. Fibrates and thiazolidinediones (pioglitazone, rosiglitazone) are hydrophobic compounds that mostly need to be administered systemically because they have poor skin penetration. Systemic dosage exposes patients to systemic side effects and requires monitoring, even though it guarantees appropriate serum and tissue concentrations. Thiazolidinedione topical formulations, such as rosiglitazone cream, have been developed; nevertheless, their clinical performance is limited, with PASI improvements of 20-30% in a small number of trials, significantly less than systemic doses [138,139]. The restriction results from the barrier role of the stratum corneum: lipophilic PPAR ligands are absorbed into the lipid matrix of the stratum corneum but are unable to enter the viable epidermis and dermis, which are home to PPAR-expressing keratinocytes, dendritic cells, and immune cells.

Nanoparticle and Liposomal Delivery

Nanoparticles (NPs) are a novel way to improve topical PPAR ligand penetration. Lipid-based NPs, such as liposomes, solid lipid NPs, and nanoemulsions, encapsulate hydrophobic PPAR ligands, lowering their melting point and surface tension and enabling stratum corneum penetration and deposition in deeper epidermal layers [140,141]. When compared to traditional cream formulations, preclinical research shows that liposomal pioglitazone or rosiglitazone formulations achieve two to five times better skin penetration and retention [38,142]. Topical NP-delivered rosiglitazone inhibited epidermal IL-17, IL-23, and TNF-α mRNA and decreased PASI-equivalent scores by around 50% in mice psoriasis models, nearing systemic dosage efficacy [143].

By manually breaching the stratum corneum barrier, microneedle-based delivery devices allow relatively large hydrophobic molecules to penetrate deeper. Early research on prototype pioglitazone-loaded microneedle patches revealed potential [144]. Topical penetration without the complexity of NPs could be facilitated by chemical modification and prodrug-based derivatization of PPAR ligands to boost water solubility while maintaining PPAR-binding activity. In dermatology, only a few of these substances have progressed to clinical development [145].

Clinical Development Status

No PPAR ligand formulation based on NPs has progressed to phase 2/3 clinical trials in psoriasis thus far, indicating a substantial unrealized translational opportunity. The regulatory uncertainty surrounding NP-based topical medications (Food and Drug Administration (FDA) guidance on nanotherapeutics is evolving), manufacturing complexity and cost-effectiveness issues, and limited corporate investment in topical psoriasis agents (biologic therapies dominate market share and development spending) are some of the obstacles to development. Developing and clinically validating NP-delivered PPAR ligands as combination partners to systemic biologics or as adjunctive topical agents for mild-to-moderate psoriasis would be a logical next step. This could lower the doses of systemic biologics needed and improve patient convenience (Table 2).

**Table 2:** Emerging drug delivery strategies. This table has been synthesized from references [38,139-145]. NP: nanoparticle; IL-23: interleukin-23; IL-17: interleukin-17; TNF-α: tumor necrosis factor-alpha; PASI: psoriasis area and severity index; mRNA: messenger RNA; PPAR: peroxisome proliferator-activated receptor

Approach	Mechanism	Evidence	Limitations
Nanoparticles	Liposomes, solid lipid NPs, nanoemulsions encapsulate hydrophobic ligands → lower melting point, better penetration	Preclinical: 2–5× better penetration; NP rosiglitazone lowers IL-17/IL-23/TNF-α mRNA, ~50% PASI-equivalent reduction in mice	No phase 2/3 trials; regulatory uncertainty; manufacturing cost
Microneedle patches	Physically breach stratum corneum and deliver hydrophobic molecules into deeper cells/tissue	Prototype pioglitazone patches show promise	Early stage of development, patient acceptability, scalability unknown
Prodrug/Derivatization	Chemical modification to increase water solubility while retaining PPAR binding	Conceptual; few compounds in dermatology pipeline	Limited clinical development
Topical formulations (creams)	Direct application	Small trials: PASI 20–30%	Inferior to systemic dosing

Synthesis, gaps, and future directions

Integrative Model: PPAR Dysregulation as a Psoriasis Nexus

The aforementioned data come together to form a cohesive model of PPAR-mediated psoriasis etiology. Balanced PPAR signaling in healthy skin preserves immunological tolerance and epidermal homeostasis: PPARα-driven lipid synthesis maintains barrier integrity, PPARγ-mediated trans-repression of NF-κB/STAT3 suppresses proinflammatory gene expression, and properly regulated PPARδ promotes keratinocyte differentiation and wound healing. PPAR-mediated metabolic control and anti-inflammatory signaling maintain systemic metabolic homeostasis and suppress autoreactive T cells in adipose tissue, liver, and immune cells. Genetic susceptibility (IL-23R loci, HLA Cw0602), environmental stressors (infections, mechanical damage, stress), and metabolic disorders (obesity, insulin resistance) all contribute to psoriasis by starting a series of events that lead to PPAR dysregulation. In suprabasal keratinocytes, elevated TNF-α, IL-1β, and NF-κB and STAT3 activation (from innate immune activation via TLR sensing or IL-23-driven Th17 expansion) directly decrease PPARγ and PPARα expression while concurrently encouraging PPARδ upregulation. This dysregulation, particular to isoforms, generates a positive feedback loop.

Loss of PPARγ-mediated trans-repression causes NF-κB/STAT3 to be derepressed. It also increases the production of IL-23, IL-17, and TNF-α, which amplifies Th17 and induces keratinocyte hyperproliferation and dendritic cell activation, further suppressing PPARγ. Loss of PPARα results in increased TEWL and decreased barrier lipogenesis. Barrier malfunction leads to exposure to pathogen-associated molecular patterns (PAMPs) and irritants, which activate NF-κB/STAT3 through TLRs and further suppress PPARα and PPARγ. Moreover, an increase in PPARδ causes an increase in anaerobic glycolysis, peroxisomal β-oxidation of barrier lipids, IL-1β/IL-17/IL-23 in suprabasal cells, and HB-EGF-mediated keratinocyte proliferation. This leads to persistent IL-17/Th17 growth, keratinocyte hyperproliferation, barrier lipid depletion, and psoriatic phenotypic amplification. MeS, IR, NAFLD, and dyslipidemia are further caused by a systemic loss of PPARγ and PPARα in adipose tissue and liver, which further depletes adiponectin and activates TNF-α/IL-6, impairing fatty acid oxidation. ATS, CVD, and psoriatic arthritis are caused by an increase in vascular inflammation and systemic IL-23/Th17 signaling. A multi-targeted strategy that addresses both cutaneous and systemic pathology can be achieved by breaking this cycle at the PPAR level through agonism (PPARγ, PPARα) or antagonism (PPARδ).

Translational gaps and clinical limitations

Despite the strong mechanistic justification, several important gaps between PPAR biology and clinical translation have been found. These include the lack of biomarker stratification; the limited understanding of cellular specificity, bioavailability, and systemic side effects; the mechanistic complexity and context-dependency; the modest clinical efficacy of monotherapy; and the underdevelopment of logical combination strategies.

Although statistically significant, PASI-75 rates of approximately 30-35% with pioglitazone monotherapy are significantly lower than those attained by contemporary biologic therapies (>80% PASI-75 with anti-IL 17, anti-IL 23 drugs). This restricts patients with biologic-refractory diseases and those with severe psoriasis. This problem is best illustrated by PPARδ, whose activation is advantageous in wound healing and some immunological populations, but its increase in psoriasiform skin appears harmful. Therapeutic strategy is complicated by this context-dependency: selective antagonism appears to be superior, but it is a novel technique requiring de novo clinical development and regulatory approval; simple agonism is probably suboptimal for PPARδ.

The majority of research looks at PPAR effects in whole tissue models or isolated cell types such as T cells, keratinocytes, and dendritic cells. It is still unclear how much PPAR signaling in fibroblasts, immune cells, and keratinocytes contributes to the overall disease phenotype. This could be explained by sophisticated spatial transcriptomics and conditional genetic models (cell-type-specific PPAR overexpression or deletion). Systemic PPAR agonism includes safety risks (bladder cancer, fracture risk with long-term pioglitazone) and metabolic side effects (weight gain, fluid retention with thiazolidinediones; myalgia and elevated liver enzymes with fibrates). Without sophisticated formulation technologies, topical administration is still less than ideal and has not yet reached clinical trials for psoriasis.

All psoriasis patients who reach a specific disease severity cutoff are currently prescribed PPAR agonists in clinical practice. However, molecular knowledge indicates that patients with *metabolically dysregulated* or *PPAR-deficient* psoriasis (defined, for instance, by higher metabolic syndrome characteristics, low baseline PPARγ expression, or a metabolic gene expression signature) may benefit more. The lack of a validated biomarker panel for PPAR-agonist response represents a precision medicine opportunity lost. Combinations with contemporary biologic agents (anti-TNF-α, anti-IL-17, and anti-IL-23) have not been thoroughly investigated, although pioglitazone + methotrexate and pioglitazone + acitretin show promise. Does the need for biologic dosages decrease with PPAR agonism? Is biologic resistance prevented or lessened by it? These are clinically pertinent concerns that need further research.

Agenda for future research

We identified several research gaps, including dual and isoform-selective agonists, PPARδ antagonism in clinical development, patient stratification by PPAR expression and metabolic phenotype, bioavailability and topical delivery innovation, mechanistic studies in humanized and patient-derived models, combination therapy randomized controlled trials, and CVD outcomes in psoriasis.

Next-generation selective agonists (high-affinity PPARγ agonists with little side effects) or glitazars (dual PPARγ/α agonists, such as MK-767, but no longer in clinical development) may be more effective than pioglitazone. Interestingly, selective regulation prevents all three PPAR isoforms from being activated simultaneously and may allow for isoform-specific therapeutic targeting (e.g., PPARγ agonism + PPARδ antagonism). In psoriasis, GSK3787 and associated selective PPARδ antagonists constitute a novel treatment class that has to be tested clinically. Phase 1/2 trials assessing topical GSK3787 or similar drugs' safety, tolerability, and effectiveness in mild-to-moderate plaque psoriasis would determine whether antagonism is a better treatment option than agonism.

The creation of a PPAR-expression and metabolic syndrome signature (e.g., lesional skin PPARγ/PPARα/PPARδ mRNA ratio, circulating metabolic biomarkers such as HOMA-IR, adiponectin, and inflammatory lipid metabolites) would make it possible to use precision-medicine techniques in which patients with low PPARγ/PPARα and metabolic comorbidities are given PPAR agonists specifically, potentially increasing response rates and reducing costs. The development and clinical validation of nanoparticle, microneedle, or chemically optimized topical PPAR ligand formulations as supplemental treatments for mild-to-moderate psoriasis should be the top priority for academic-industry collaborations. Success would lessen the need for systemic medications with metabolic side effects and increase the therapeutic toolset.

The bench-to-clinic gap would be filled by patient-derived 3D skin equivalents with psoriatic genetic backgrounds (e.g., modified to express psoriasis-risk single-nucleotide polymorphisms (SNPs)) treated with PPAR ligands. The cell types and pathways that are PPAR-responsive would be made clear by single-cell RNA-seq of psoriatic lesional skin before and after PPAR agonist treatment (such as pioglitazone in a phase 2 trial). Compared to murine models, humanized mouse models such as non-obese diabetic (NOD) severe combined immune deficiency (SCID) IL-2 receptor gamma chain null (IL2rγ^null^) mutation mice (NSG®) that block signaling, destroying NK cell function, engrafted with human psoriatic skin and immune cells, may more accurately represent human PPAR biology. Pioglitazone + biologic (e.g., pioglitazone + secukinumab (IL-17 inhibitor)) in moderate-to-severe psoriasis to evaluate long-term results, impact on biologic sparing, and additive efficacy. PPARγ agonist combined with PPARδ antagonist in a preclinical model to see if the combination offers PPARγ-mediated advantages while addressing the harmful PPARδ overexpression.

Large, prospective observational studies or pragmatic randomized controlled trials assessing whether PPAR agonism (especially pioglitazone or future agents) improves vascular inflammation and lowers cardiovascular events in psoriasis patients would establish cardiometabolic benefits beyond skin improvement, potentially expanding clinical indication.

## Conclusions

Psoriasis is increasingly recognized as a systemic inflammatory‑metabolic disorder rather than a purely cutaneous disease. Central to this paradigm is dysregulation of PPARs, nuclear transcription factors that integrate metabolic and immune signaling. Isoform‑specific dysfunction contributes to disease pathogenesis: loss of PPARγ in adipose and immune cells promotes MeS and systemic inflammation, while its downregulation in skin removes repression of NF‑κB/STAT3, enabling unchecked IL‑23/Th17 signaling and keratinocyte hyperproliferation. Reduced PPARα impairs epidermal lipogenesis, weakening barrier integrity and perpetuating irritant‑driven inflammation. Conversely, PPARδ overexpression drives keratinocyte proliferation via HB‑EGF and reprograms metabolism toward glycolysis and lipid β‑oxidation, amplifying psoriatic pathology. Therapeutically, pioglitazone (PPARγ agonist) demonstrates moderate efficacy and favorable metabolic effects, but its slow onset and side‑effect profile limit widespread adoption. Fibrates (PPARα agonists) remain underexplored despite a strong mechanistic rationale for barrier repair, while PPARδ antagonism shows promising preclinical activity. Translational progress requires next‑generation ligands with improved isoform selectivity and pharmacokinetics, rational combination strategies integrating PPAR modulation with biologics or conventional agents, and innovative topical delivery systems (NPs, microneedles, prodrugs) to overcome bioavailability barriers. Future clinical trials should assess long‑term safety, cardiometabolic outcomes, and patient stratification by metabolic phenotype. By simultaneously addressing cutaneous inflammation and systemic metabolic dysfunction, the *psoriatic march*, PPAR modulation offers a dual‑benefit therapeutic approach. Coordinated development could reshape treatment paradigms, positioning PPAR‑targeted therapies as adjuncts or complements to biologics in the broader management of psoriatic disease.
